# Penalty Dynamic Programming Algorithm for Dim Targets Detection in Sensor Systems

**DOI:** 10.3390/s120405028

**Published:** 2012-04-18

**Authors:** Dayu Huang, Anke Xue, Yunfei Guo

**Affiliations:** 1 School of Information Science & Engineering, East China University of Science & Technology, Shanghai 200237, China; 2 Institute of Information and Control, Hangzhou Dianzi University, Hangzhou 310018, China; E-Mails: akxue@hdu.edu.cn (A.X.); gyf@hdu.edu.cn (Y.G.)

**Keywords:** track-before-detect, sensor systems, signal-to-noise-ratio, dynamic programming, maneuvering, multi-target tracking, feedback

## Abstract

In order to detect and track multiple maneuvering dim targets in sensor systems, an improved dynamic programming track-before-detect algorithm (DP-TBD) called penalty DP-TBD (PDP-TBD) is proposed. The performances of tracking techniques are used as a feedback to the detection part. The feedback is constructed by a penalty term in the merit function, and the penalty term is a function of the possible target state estimation, which can be obtained by the tracking methods. With this feedback, the algorithm combines traditional tracking techniques with DP-TBD and it can be applied to simultaneously detect and track maneuvering dim targets. Meanwhile, a reasonable constraint that a sensor measurement can originate from one target or clutter is proposed to minimize track separation. Thus, the algorithm can be used in the multi-target situation with unknown target numbers. The efficiency and advantages of PDP-TBD compared with two existing methods are demonstrated by several simulations.

## 1. Introduction

For surveillance applications, a sensor system can be installed to detect and track targets within a given region [1]. The sensor system can provide the data originating from targets, clutter or noise. In order to detect targets and estimate their trajectories, the sensor data should be processed. Traditional strategies use a detect-then-track approach that declares the measurements when the sensor data exceeds a threshold (hard decisions are made for each scan data), and uses these measurements to estimate target trajectories using tracking algorithms. This is acceptable when the signal-to-noise-ratio (SNR) is high. However, for low SNR targets (e.g., stealth aircraft), the signal amplitudes reflected from the targets might not be strong enough to be detected. Therefore, the targets may be buried in the clutter and noise. One possible approach is to lower the threshold to avoid too many missing tracks, but a low threshold would give a high rate of false detections [2–4]. On the other hand, track-before-detect (TBD) techniques make soft decisions on the sensor data. Unlike the traditional approaches, TBD detections are not declared at each scan. Instead, a number of scans of data are processed, then the estimated target track is acquired when the detection is declared [5]. Because they use more sensor data, TBD techniques are efficient for detecting low SNR targets in sensor systems.

Previously developed techniques for TBD include Hough transform [6], dynamic programming [7,8] and particle filter [9,10], *etc.* DP-TBD is efficient for dim target detection and it facilitates engineering applications, so recently it has received more and more attention [5,11–13]. In [11], the authors derive a power-efficient TBD-based algorithm for early detection in sensor systems, and the proposed method is based on a generalized likelihood ratio test approach. The detection and tracking performance of DP-TBD algorithm is analyzed in [5,12]. It shows that the tracking performance is poor, even though the detection performance is good, and the track separation has a bad influence on the tracking performance. In [13], a modified DP algorithm is presented to minimize the track separation by using a tracking approach.

However, traditional DP-TBD methods perform poorly for maneuvering target detection and tracking. In [14], a modified DP-TBD algorithm, which uses state prediction to change the transition step, is developed to track a turning target. Some other TBD algorithms have the abilities to detect and tracking maneuvering targets. For example, particle filter combined with multi-model can be applied to detect and track a maneuvering target, but the computation cost is high [15]. Interacting multi-model probabilistic data association with amplitude feature (IMMPDAF-AI) is another alternative approach, which makes tracking decisions by assessing the probabilities of target models to provide rapid and accurate decisions for both true track acceptance and false track dismissal in track formation [16].

The above TBD algorithms consider only a single target, whereas many practical situations require the detection of multiple targets [2]. In [17–19], a particle filter TBD is proposed to deal with a two non-maneuvering targets split situation, but with the assumption that the maximum number of targets is known. Traditional DP-TBD methods consider only a single target with slowly maneuvering motion [2,12], when target maneuvers or multiple targets appear in the surveillance region, the performance will fall rapidly.

In this paper, a modified algorithm (PDP-TBD) is proposed to detect and track multiple maneuvering dim targets of unknown target number in sensor systems. For PDP-TBD, the performances of tracking techniques are used as a feedback to the detection part. Thus, the algorithm combines traditional tracking techniques with DP-TBD and its performance of detection and tracking can be improved. Two technical modifications are proposed in this paper, which are described as follows.

1. The proposed algorithm uses the performances of tracking techniques as a feedback to the detection part, and the feedback is constructed by a penalty term in the merit function. The penalty term is a function of the possible target state estimation, which is obtained by traditional tracking techniques. If the tracking techniques perform well (the estimation errors are small), the target will have high influence on the merit function. For a larger merit function is more likely to be originated from the target [5,12], the target will be more likely to be detected. If the tracking techniques perform very poorly (the estimation errors are big), the penalty term will have almost no influence on the merit function. Thus, PDP-TBD will be equivalent to DP-TBD, *i.e.*, DP-TBD can be seen as a special case of PDP-TBD. With this feedback, the detection performance can be improved and the influence of the clutters can be reduced, and the tracking accuracy can also be improved. Though the tracking techniques are based on interacting multiple model (IMM) to deal with target maneuver situation, they can be adopted by other proper tracking methods.
2. The track separation phenomenon has a bad influence on the tracking accuracy and target number estimation [5,12]. When detecting and tracking two targets which are not well separated (e.g., the targets have crossing trajectories), it is easy to lose one target using DP-TBD. To solve this problem, a reasonable constraint that a sensor measurement can originate from only one target or clutter is used in this paper. Based on the constraint, a new step called repetition is added to the procedure of PDP-TBD. With this modification, the track separation can be minimized and the new algorithm can be applied to detect multiple targets.

The outline of the paper is as follows: in Section 2, the target model and the sensor measurement model are formulated. Section 3 gives the description of the merit function with a penalty term. The procedure of PDP-TBD algorithm is given in Section 4. The simulation results and conclusions are presented in Section 5 and 6, respectively.

## 2. Problem Formulation

We consider here the problem of detecting and tracking multiple dim point targets in sensor systems. Suppose that there are *N* ≥ 1 targets (*N* is unknown) moving in x-y plane. The target motion models of each target can be written in the form:

(1) $${\text{s}}_{k+1}=f({\text{s}}_{k},{\text{v}}_{k})$$

where *k* is the discrete time index, *k* = 1, 2…, *K*, and *K* is the total scan number. ***s**_k_* is the target state and *f* (·) is the system dynamics function. The vector ***v**_k_* is zero-mean white Gaussian noise sequences with a covariance matrix ***Q***.

In practical applications, the target can be various aircrafts, such as airplanes, helicopters or missiles, and the sensor can be radar, sonar, *etc.* When these targets move into a surveillance region, the sensor provides the data originated from the targets or clutters. In this section, for simplicity, the sensor systems are assumed to produce a matrix of 2-dimensional x-y target position along with the target reflected power. Suppose that there are *m*(*k*) sensor measurements received at scan *k*, which are originated from the targets and clutters. The sensor measurement set at scan *k* can be denoted as:

(2) $$\text{Z}(k)=\left\{{\text{z}}_{k}(i)=\left({\text{z}}_{P,k}(i),z_{I,k}(i)\right)|i=1,2,\cdots m(k)\right\}$$

where ***z****_P,k_* (*i*) = (*z_x,k_* (*i*),*z_y,k_* (*i*)) is the *i*^th^ position measurement and *z_I,k_* (*i*) represents the reflected power of ***z****_P,k_* (*i*), denoted as ***z****_I,k_* (*i*) = *g* (***z****_P,k_* (*i*)).

If the *i*^th^ sensor measurement ***z****_k_* (*i*) is originated from the targets, ***z****_P,k_* (*i*) can be represented by:

(3) $${\text{z}}_{P,k}(i)=\text{H}{\text{s}}_{k}+{\text{w}}_{k}$$

where ***H*** is a measurement matrix. The noise vector ***w****_k_* is zero-mean white Gaussian sequences and independent of ***v**_k_*. The covariance matrix of ***w****_k_* is ***R***.

If the *i* th sensor measurement ***z****_k_*(*i*) is originated from the clutters, ***z****_P,k_*(*i*) is assumed to be uniformly distributed in the surveillance region. The reflected power amplitude probability density function *p_I_*(·) is modeled as a Rayleigh random variable described in [15,16].

Target-originated measurements:

(4) $$p_{I}\left(z_{I,k}(i)\right)=\frac{z_{I,k}(i)}{\left(1+d_{\text{SNR}}\right)}\exp\left(-\frac{z_{I,k}{\left(i\right)}^{2}}{2(1+d_{\text{SNR}})}\right)$$

Clutter-originated measurements:

(5) $$p_{I}\left(z_{I,k}(i)\right)=z_{I,k}(i)\exp\left(-\frac{z_{I,k}{\left(i\right)}^{2}}{2}\right)$$

where exp (·) is the exponential function and *d_SNR_* is the expected SNR of target returns.

The problem of multi-target detection and tracking is as follows. For a given sensor measurement sequence of *K* scans, we wish to determine the trajectories (state sequences) most likely to have originated from the actual targets and estimate the actual target number.

## 3. Merit Function with a Penalty Term

Traditional DP-TBD methods integrate the measurements along possible target trajectories, returning as possible targets those trajectories for which the merit function exceeds a threshold. However, it is proposed based on a single target with slowly maneuvering motion [2].

In this Section, a new algorithm named PDP-TBD is proposed to detect and track multiple maneuvering dim targets. By integrating a penalty term into the merit function, the algorithm combines traditional tracking techniques with DP-TBD. With this modification, the new algorithm has the advantages of the tracking techniques for different target motions.

### 3.1. Traditional Merit Function Description

For DP-TBD, ***X****_k_* represents the set of all possible states of the targets at scan *k*. ***x****_k_* is a possible state of ***X****_k_* and ***x****_k_* ∈ ***X****_k_*. *I*(***x****_k_*) represents the merit function of ***x****_k_*. Equation (6) is used to estimate the trajectories most likely to have originated from the actual targets.

For each ***x****_k_*, to find:

(6) $$\left\{{\hat{\text{x}}}_{K}\right\}=\left\{{\text{x}}_{K}:I({\text{x}}_{K})>V_{T}\right\}$$

where *I*(***x****_K_*) is the final stage of merit function and *V_T_* is the threshold. *K* is the total scan number. By Equation (6), the estimated trajectories are those trajectories (state sequences) for which the merit function exceeds the threshold *V_T_*.

The construction of the merit function is a key problem for DP-TBD. One approach is to use a likelihood-ratio function in the merit function [8]. The likelihood-ratio function is obtained by taking into account the statistical models of background signal and noise, hence, the merit function needs the prior knowledge of signal and noise.

For another approach, the merit function is calculated only depending on the reflected power, therefore, no knowledge of the signal and noise statistics is required [5]. For all ***x****_k_* ∈ ***X****_k_* and ***x****_k_*_−1_ ∈ ***X****_k_*_−1_ the merit function is given by:

(7) $$I({\text{x}}_{k})=\underset{{\text{x}}_{k-1}\in \text{PRE}({\text{x}}_{k})}{\max}[I({\text{x}}_{k-1})]+g({\text{x}}_{k})$$

where *PRE*(***x****_k_*) represents the state transition range of ***x****_k_*. ***I***(***x****_k_*_−1_) and ***I***(***x****_k_*) are the merit functions of ***x****_k_*_−1_ and ***x****_k_* respectively. *g*(***x****_k_*) is the reflected power of ***x****_k_*. Equation (7) indicates that the maximization is performed over the ***x****_k_*_−1_ for which a transition to ***x****_k_* is possible and the merit function is a sum of the reflected power. For very low SNR environment, the clutter is likely to be detected as the target, thus the actual target may be lost and the performance of DP-TBD may fall. For maneuvering targets detection and tracking, the state transition range should be expanded and the computation cost will increase.

### 3.2. Merit Function with a Penalty Function

PDP-TBD uses the tracking performances as a feedback to the detection part, and the feedback is constructed by a penalty term in the merit function. Then for all ***x****_k_* ∈ ***X****_k_* and ***x****_k_*_−1_ ∈ ***X****_k_*_−1_, the new merit function is designed as:

(8) $$I({\text{x}}_{k})=\underset{{\text{x}}_{k-1}\in \text{PRE}({\text{x}}_{k})}{\max}[I({\text{x}}_{k-1})+Pe({\hat{\text{x}}}_{k|k},{\text{x}}_{k})]+g({\text{x}}_{k})$$

where ***x̂***_*k*∣*k*_ is the possible target estimation of ***x****_k_* and it is obtained by traditional tracking methods. Compared with Equation (7), a penalty term *Pe*(***x̂***_*k*∣*k*_, ***x****_k_*) is added to the new merit function. *Pe*(***x̂***_*k*∣*k*_, ***x****_k_*) is a function of ***x̂***_*k*∣*k*_ and ***x****_k_*. Its value is influenced by the Minkowski distance between ***x̂***_*k*∣*k*_ and ***x****_k_*. *g*(***x****_k_*) is the reflected power of ***x****_k_*. The new merit function consists of a penalty term *Pe*(***x̂***_*k*∣*k*_, ***x****_k_*) and an amplitude term *g*(***x****_k_*). For the likelihood-ratio function is not included in the merit function, the algorithm can be used without any prior knowledge of signal and noise. It may be useful if the statistics of signal and noise are difficult to obtain.

By the penalty term, the feedback of the tracking performances is constructed and traditional tracking techniques are combined with DP-TBD. If the tracking techniques perform well (the estimation errors are small), the penalty term will be small for clutter and big for targets. Therefore, the target will have much higher influence on the penalty term than the clutter. For a larger measurement is more likely to have originated form the target than a smaller measurement[5,12] (*i.e.*, a larger merit function is more likely to be originated from the target than a smaller function), the probability of detecting the target can be improved through the penalty term. If the performances of tracking techniques degrade (the estimation errors are increasing), the target will have less influence on the penalty term than the clutter, thus, the performance of PDP-TBD will degrade. If the tracking techniques perform very poorly (*i.e.*, the estimation errors are very big and the possible state estimation ***x̂***_*k*∣*k*_ is far from ***x****_k_*), the penalty term will be very small, and it may even be nearly equal to zero in the merit function. Thus, the penalty term will have almost no influence on the merit function and the algorithm will be equivalent to DP-TBD. As described above, the performance of PDP-TBD relies on the efficiency of the tracking techniques, but in any case, PDP-TBD performs better than DP-TBD by constructing the new merit function. And with the penalty term, the detection performance can be improved and the influence of the clutters can be reduced. Meanwhile, the tracking accuracy can also be improved.

For the efficiency of the feedback relies on the performances of tracking techniques, the proper tracking methods should be chosen according to the target motions. In this paper, PDP-TBD is applied to detect and track maneuvering targets in sensor systems, hence, the tracking techniques used here are based on IMM [20,21]. For multi-target situation, the data association methods such as joint probability data association (JPDA) [22] and multiple hypothesis testing (MHT) [23] can be used. By combining the IMM methods with DP-TBD, the new algorithm can be extended for maneuvering targets. For different tracking methods can be adopted for PDP-TBD according to the target motions, the application scope of the algorithm can be significantly extended compared to traditional DP-TBD methods.

**Figure 1.**
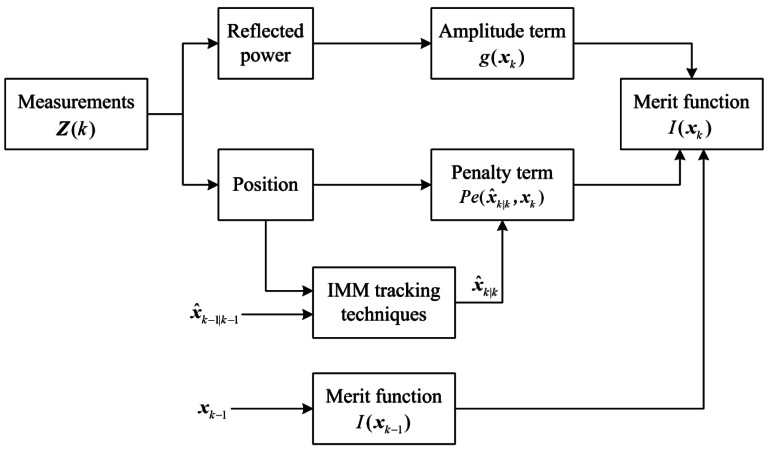
Merit function calculation block diagram at scan *k*.

Suppose that the transition from ***x****_k_*_−1_ to ***x****_k_* is possible, the block diagram of merit function calculation is shown in Figure 1. The sensor measurement set ***Z***(*k*) originated from the targets or clutters consists of the position measurements and reflected power. When calculating the merit function, the amplitude term *g*(***x****_k_*) is the reflected power and the penalty term *Pe*(***x̂***_*k*∣*k*_, ***x****_k_*) is a function of ***x****_k_* and ***x̂***_*k*∣*k*_. ***x̂***_*k*∣*k*_ is obtained by IMM tracking techniques. Then the merit function *I*(***x****_k_*) at scan *_k_* is a sum of *I*(***x****_k_*_−1_), penalty term *Pe*(***x̂***_*k*∣*k*_, ***x****_k_*) and amplitude term *g*(***x****_k_*).

## 4. PDP-TBD Algorithm

DP-TBD is a modified version of the Viterbi algorithm. It is equivalent to an exhaustive search of all possible target trajectories, returning all state sequences for which the final stage merit function exceeds a specific threshold [12]. DP-TBD includes the following steps: initialization, recursion, termination and backtracking.

For DP-TBD, when calculating the merit function using Equation (7), the maximization is performed over the ***x****_k_*_−1_ for which a transition to ***x****_k_* is possible. This is a reason which results in the track separation, and when two targets are not well separated, one target state may be lost using Equation (7). Thus, DP-TBD cannot be applied to deal with multi-target situations. To solve this problem, a reasonable constraint that a sensor measurement can originate from only one target or clutter is proposed. Based on this constraint, a step called repetition is added to PDP-TBD.

### 4.1. PDP-TBD Procedure

**Figure 2.**
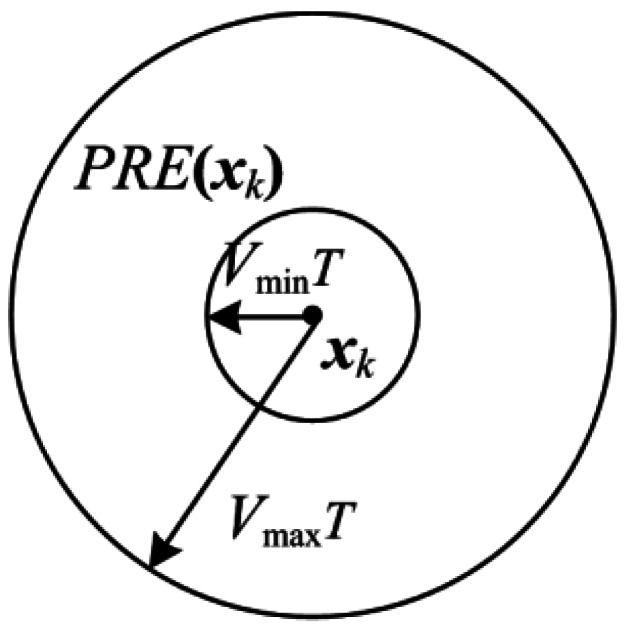
State transition range.

Let ***X****_k_* represent the set of all possible states of the targets at scan *k*. ***x****_k_* is a possible state of ***X****_k_, i.e.*, ***x****_k_* ∈ ***X****_k_*. The state transition range *PRE*(***x****_k_*) used in PDP-TBD is shown in Figure 2. In Figure 2, *T* is the time interval between successive scans, and [*V*_min_, *V*_max_] is the range of target velocity.

*I*(***x****_k_*) is the merit function of ***x****_k_*. *g*(***x****_k_*) is the reflected power and it is given by the sensor systems. The state which is associated with ***x****_k_* at last scan is stored in ***ψ****_xk_* (*K*). The PDP-TBD algorithm is carried out as follows:

Step 1: initialization. *K* = 1, for all ***x***_1_ ∈ ***X***_1_,

(9) $$I({\text{x}}_{1})=g({\text{x}}_{1}),{\text{ψ}}_{{\text{x}}_{1}}(1)=0$$

Step 2: recursion. 2 ≤ *k* ≤ *K*, for all ***x****_k_* ∈ ***X****_k_*,

(10) $$I({\text{x}}_{k})=\underset{{\text{x}}_{k-1}\in \text{D}}{\max}[I({\text{x}}_{k-1})+Pe({\hat{\text{x}}}_{k|k},{\text{x}}_{k})]+g({\text{x}}_{k})$$

(11) $${\text{ψ}}_{{\text{x}}_{k}}(k)=\underset{{\text{x}}_{k-1}\in \text{D}}{\arg\max}[I({\text{x}}_{k-1})+Pe({\hat{\text{x}}}_{k|k},{\text{x}}_{k})]$$

where D is the valid range and D = *PRE*(***x****_k_*) here. The merit function *I*(***x****_k_*) consists of three parts: *I*(***x****_k_*_−1_), *Pe*(***x̂***_*k*∣*k*_, ***x****_k_*) and *g*(***x****_k_*). *I*(***x****_k_*_−1_) is the merit function at scan *k*−1 and *Pe*(***x̂***_*k*∣*k*_, ***x****_k_*) is the penalty term. In this paper, the calculation of *Pe*(***x̂***_*k*∣*k*_, ***x****_k_*) is as follows:

(12) $$Pe({\hat{\text{x}}}_{k|k}|{\text{x}}_{k})=\{\begin{array}{cc} \frac{\alpha }{\left\|\text{H}{\hat{\text{x}}}_{k|k}-\text{x}{\text{p}}_{k}\right\|} & {\hat{\text{x}}}_{k|k}\neq 0,{\text{x}}_{k-1}\in \text{D}\\ 0 & \text{others} \end{array}$$

where ***xp****_k_* is the position of ***x****_k_* and ***H*** is a measurement matrix. *α* is a scale factor which is affected by the size of measurement noise and the estimation errors. ***x̂***_*k*∣*k*_ is the estimation of ***x****_k_* and it is obtained by the tracking techniques. ***x̂***_*k*∣*k*_ = 0 represents no estimation being obtained for ***x****_k_*. $\left\|\text{H}{\hat{\text{x}}}_{k|k}-\text{x}{\text{p}}_{k}\right\|$ represents the Minkowski distance between ***Hx̂***_*k*∣*k*_ and ***xp****_k_*.

Step 3: repetition. The track separation is a key problem for DP-TBD algorithm [5,12]. It produces a large number of false trajectories, thus it reduces the tracking accuracy and causes errors in estimating the target number. When two targets are not well separated (e.g., the two targets have crossing trajectories), it is easy to lose one target. Considering the track separation problem, Step 3 is added to the procedure of PDP-TBD.

Regardless of the resolution influence and some other factors, we suppose that a sensor measurement cannot be originated by two targets. Then a reasonable constraint is proposed in Step 3 to eliminate the track separation.

Constraint: a sensor measurement can originate from one target or clutter, *i.e.* a measurement can have only one source.

Therefore, a possible state of the target can transit to only one state in the next scan, *i.e.* no possible states of the target at scan *k* can be associated with a same state at scan *k*−1. The constraint is a classic constraint for traditional tracking algorithm, such as JPDA [22,24]. The procedure of Step 3 is given as follows: if several possible states share a same associated state after Step 2 at scan *k*, the possible state with the highest merit function will have “the first priority”, and the other states will repeat Step 2 again by excluding the same associated state stored from the valid range *D*. Next, if some possible states still share a same associated state, the above repetition will be processed again. The repetition will not be terminated until no possible states share a same associated state (the constraint is satisfied).

**Figure 3.**
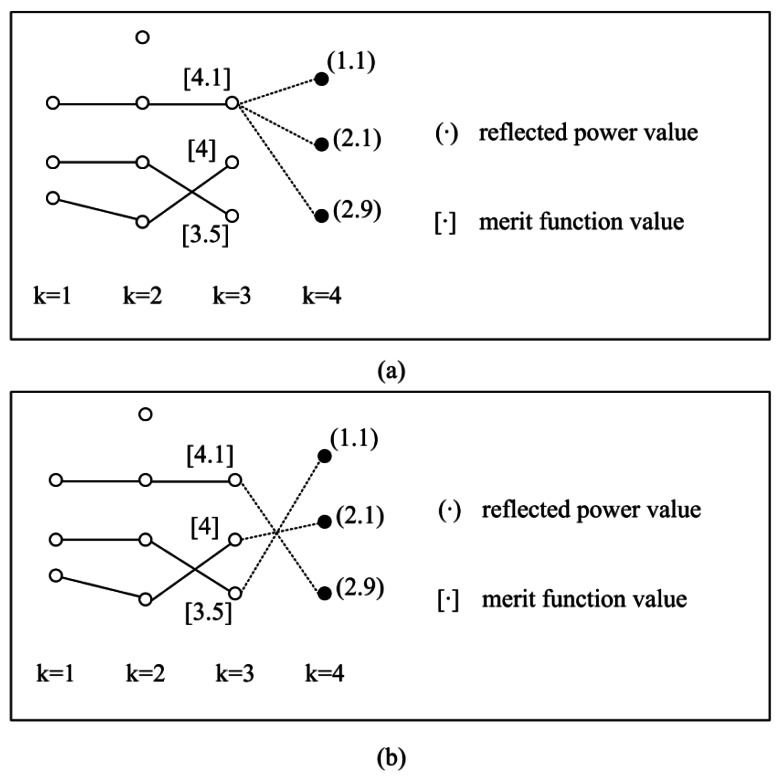
An example (**a**) After Step 2 (track separation exists); (**b**) After Step 3 (the constraint is satisfied).

The times of repetition is influenced by the clutter density. If the clutter number is below 200 in the surveillance region of 10 km × 10 km, the repetition will be terminated within six cycles. An example is given in Figure 3. Suppose that there are three possible states obtained at scan *K* = 3 and the three possible states are all in state transition ranges. Figure 3(a) shows the track separation phenomenon after Step 2, *i.e.*, only the single best state is retained after Step 2, while all other states are discarded. Inspection of Figure 3(a) shows that the three possible states at scan *K* = 4 are associated with a same state at scan *K* = 3. When two of the three states are originated from the targets (*i.e.*, two targets exist), according to the track separation shown in Figure 3(a), one target is lost. Figure 3(b) shows the new associated states after Step 3 (the constraint is satisfied and no target is lost).

Although the above constraint is used in Step 3, the clutter and target measurements may coincide with practical application. Thus, after Step 3, the situation that no state is associated with a state at next scan may exist. And if this state is originated from the target, the target will be probably lost. Considering this situation, if no state is associated with a state at next scan after Step 3, the possible state estimation obtained will be used as the associated state.

Furthermore, although PDP-TBD can be applied to deal with multi-target situations by Step 3, it may be worse when the preferred association is not the one that best matches the past target dynamics. To solve this problem, a penalty term is constructed to combine DP-TBD with traditional tracking methods, which is described in Section 3.2. According to Section 3.2, if the tracking methods work properly, the target will be much more likely to be detected than the clutter. Hence, the disadvantage by using Step 3 can be greatly alleviated.

Step 4: Termination and backtracking. For the final stage merit function of PDP-TBD consists of a penalty term, the threshold is difficult to be determined. In this step, a decision function is applied to replace the final stage merit function when determining the target trajectories. The decision function is a sum of reflected power. Therefore, the threshold can be determined without influence of the penalty term. For all ***x****_k_* ∈ ***X****_K_*, for *k* = *K, K*−1, …, 2:

(13) $${\text{x}}_{k-1}={\text{ψ}}_{{\text{x}}_{k}}(k)$$

All possible trajectories (state sequences) of the targets are obtained by Equation (13). Let ***Tra*** represent all possible trajectories of the targets. The detection function is a sum of reflected power of each possible trajectory. For possible trajectory {***x̃***_1_, ***x̃***_2_, …, ***x̃****_K_*} ∈ ***Tra***, It is given by:

(14) $$Id({\tilde{\text{x}}}_{K})=\sum_{k=1}^{K}g({\tilde{\text{x}}}_{k})$$

Termination: for all *Id*(***x̃****_K_*) and threshold *V_T_*, find:

(15) $$\left\{{\hat{\text{x}}}_{K}\right\}=\left\{{\tilde{\text{x}}}_{K}:Id({\tilde{\text{x}}}_{K})>V_{T}\right\}$$

Backtracking: for all ***x̃****_K_, k* = *K, K*−1, …, 2:

(16) $${\hat{\text{x}}}_{k-1}={\text{ψ}}_{{\hat{\text{x}}}_{k}}(k)$$

The trajectories are recovered using Equations (15) and (16), and the number of the recovered trajectories is the estimated target number.

### 4.2. Penalty Term Calculation Using Tracking Techniques

**Figure 4.**
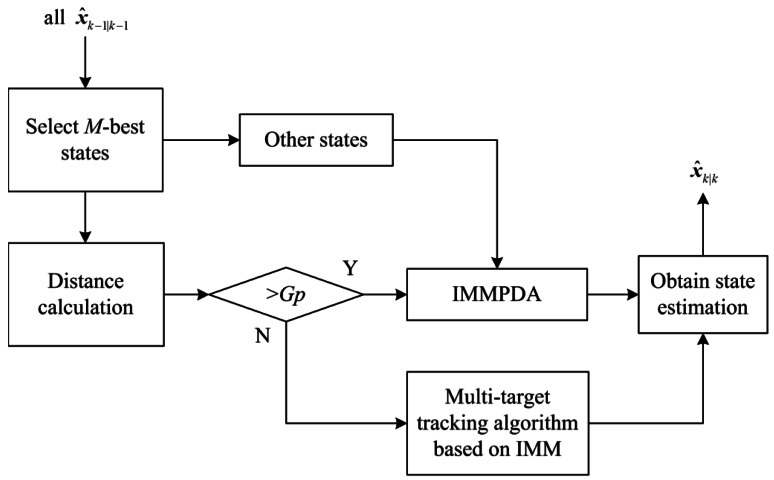
Tracking techniques block diagram.

When calculating the penalty term using Equation (12), ***x̂***_*k*∣*k*_ is essential and it is obtained by the tracking techniques. For the tracking techniques, state initiation is referred to [25] in this paper. Figure 4 shows the block diagram of the tracking techniques after state initiation, and it is the detailed process of the “IMM tracking techniques” block in Figure 1.

In Figure 4, at scan *k*, the estimations of the *M* -best states are selected from all state estimations ***x̂***_*k*−1∣*k*−1_ which are obtained at scan *k*−1. The *M* -best states are the states with the *M* -highest merit function. Next, the Minkowski position distance *G*_distance_ of the *M* selected state estimations are calculated. *Gp* is used as a threshold to determine whether the selected states are close. If, *G*_distance_ > *Gp* IMMPDA [22] will be chosen to estimate the selected states, otherwise IMM multi-target tracking algorithm such as IMMJPDA and IMMMHT will be chosen. IMMPDA is also applied to estimate the unselected states. Then all possible state estimations of the targets at scan *k* can be obtained.

IMMMHT is chosen as IMM multi-target tracking algorithm in Figure 4. It is a combination of IMM method [26] and MHT described in [23,27]. For the IMMMHT used here is applied to track multi-target, we only need to consider track maintenance.

PDA is efficient and its computation cost for tracking a single target is small [22]. JPDA and MHT are more efficient for multi-target tracking, but their computation cost is much higher than PDA. In this paper, the *M*-best selected states estimations can be obtained more accurately by multi-target tracking methods. It guarantees the estimation errors of the *M* most likely target states (the *M*-best states) to be small. Meanwhile, the computation cost can be reduced.

## 5. Simulation Results

In practical application, the CV (Constant velocity) and CT (Coordinate turn) models are two of the most common forms of target motion in the Cartesian plane [16]. An IMM method consisting of CV and CT models can be used to cover a wide range of maneuvers [28], for example, an aircraft makes cruising flight. Therefore, in this simulation, CV and CT models are used to describe the target maneuvering moving motion. The target state is defined as ***s**_k_* = [x(*k*), x˙(*k*), y(*k*), y˙(*k*), *ω*(*k*)]*^T^*, where (x(*k*), y(*k*)) and (x˙(*k*), y˙(*k*)) denote the target position and velocity in x-y plane respectively. *ω*(*k*) is the turn rate. The system dynamics function *f*(·) is given by:

For the CV model:

(17) $$f({\text{s}}_{k},{\text{v}}_{k})=\left[\begin{array}{ccccc} 1 & T & 0 & 0 & 0\\ 0 & 1 & 0 & 0 & 0\\ 0 & 0 & 1 & T & 0\\ 0 & 0 & 0 & 1 & 0\\ 0 & 0 & 0 & 0 & 0 \end{array}\right]{\text{s}}_{k}+\left[\begin{array}{ccc} 0.5T^{2} & 0 & 0\\ T & 0 & 0\\ 0 & 0.5T^{2} & 0\\ 0 & T & 0\\ 0 & 0 & 0 \end{array}\right]{\text{v}}_{k}$$

For the CT model:

(18) $$f({\text{s}}_{k},{\text{v}}_{k})=\left[\begin{array}{ccccc} 1 & \frac{\sin\left(\omega (k)T\right)}{\omega (k)} & 0 & -\frac{1-\cos\left(\omega (k)T\right)}{\omega (k)} & 0\\ 0 & \cos\left(\omega (k)T\right) & 0 & -\sin\left(\omega (k)T\right) & 0\\ 0 & \frac{1-\cos\left(\omega (k)T\right)}{\omega (k)} & 1 & \frac{\sin\left(\omega (k)T\right)}{\omega (k)} & 0\\ 0 & \sin\left(\omega (k)T\right) & 0 & \cos\left(\omega (k)T\right) & 0\\ 0 & 0 & 0 & 0 & 1 \end{array}\right]{\text{s}}_{k}+\left[\begin{array}{ccc} 0.5T^{2} & 0 & 0\\ T & 0 & 0\\ 0 & 0.5T^{2} & 0\\ 0 & T & 0\\ 0 & 0 & T \end{array}\right]{\text{v}}_{k}$$

where *T* is the time period between successive scans. Suppose that the noise variances of ***w***_k_ in each coordinate are equal, the measurement noise covariance matrix is denoted as ***R*** = *diag*(*σ*^2^, *σ*^2^). The measurement matrix in Equation (3) is given by:

(19) $$\text{H}=\left[\begin{array}{ccccc} 1 & 0 & 0 & 0 & 0\\ 0 & 0 & 1 & 0 & 0 \end{array}\right]$$

The surveillance region covers an area of 10,000 m on x axes and 10,000 m on y axes. At each scan, the number of clutters is Poisson distributed with parameter *λ*, where *λ* is the average number of clutters. The scan period is *T* = 1 s and the total frames processed is *K* = 20. The measurement noise covariance matrix is ***R*** = *diag*(900,900). For PDP-TBD,*V*_max_ = 400m/s, *V*_min_ = 0m/s and the threshold is *V_T_* = 30. The Euclidean distance is used in Equation (12), which is a special case of the Minkowski distance. And the scale factor *α* in Equation (12) is *α* = 3*σ* = 90. Considering the computation cost of the algorithm, *M* = 3.

The performance of PDP-TBD is compared against IMMPDAF-AI and DP-TBD [5] for multiple maneuvering targets detection and tracking in different target SNR values and clutter densities. In this section, it should be noted that IMMPDAF-AI is different from IMMPDA. IMMPDA is a traditional tracking algorithm which uses detect-then-track approach and cannot be applied to detect dim targets. On the other hand, IMMPDAF-AI can be seem as an extension of IMMPDA. It combines the advantageous concept of target amplitude from classical multiple track detection and the robustness of IMMPDA technique for recursive track formation in clutter and extend to perform track maintenance [16]. Thus, it can be applied to track before detect dim targets. In this section, IMMPDAF-AI is applied to 2-dimensional x-y surveillance region with track formation and track maintenance unified. And in the simulations, we use IMMPDAF-AI rather than IMMPDA when comparing these algorithm performances. For DP-TBD in the simulation comparison, we delete trajectories which share above 10 common states with a trajectory of lower merit function [5,12].

In this section, the probability of correct target number estimation is used to illustrate the detection performance. For example, two targets appear in the surveillance region, after 200 runs, if two targets are declared in 150 runs, the probability of correct target number estimation will be calculated as 150/200 = 75%. After the target number is declared, we use correct track probability to illustrate the tracking accuracy. Considering the difference of the three algorithms, for PDP-TBD and DP-TBD, when the detected position state is equal to the state originated from the actual target, the detected state is correctly tracked. For IMMPDAF-AI, when the estimated error between the detected position state (*x̂, ŷ*) and the actual target state (*x, y*) is smaller than 2*σ*:

(20) $$\begin{array}{cc} \left|\hat{x}-x\right|<2\sigma , & \left|\hat{y}-y\right|<2\sigma \end{array}$$

We consider the estimated state is correctly tracked. Therefore, correct track probability is calculated as the rate of correct tracks over all tracks. For example, there are 400 target states obtained, if 360 states are correctly tracked using PDP-TBD, the correct track probability will be calculated as 360/400 = 90%.

### 5.1. Scenario-1

Two targets appear in the surveillance region for the first scenario. The target initial states are [4500, −100,6000, −200,0.15] and [2000,150,5000,100,0.12] with assumed positions in meters, velocities in m/s and turn rates in rad/s. Target 1 makes an approximate CT motion when *k* = 1 ∼ 15s, and rest of the scans are approximate CV motions. Target 2 makes approximate CT motion. The two targets are well separated in this scenario.

**Figure 5.**
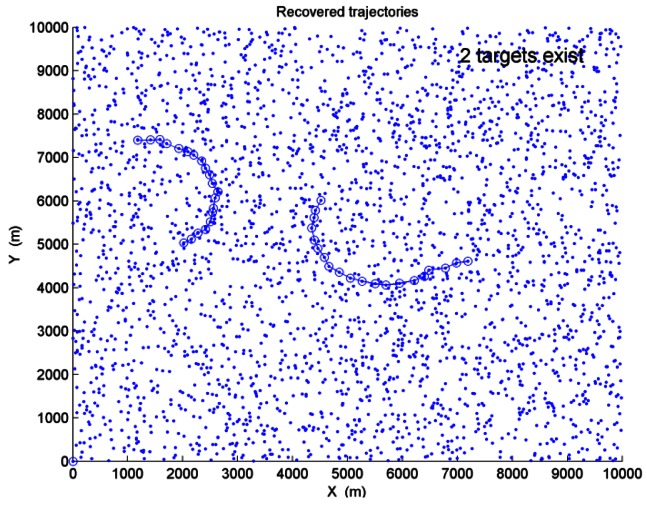
Recovered trajectories and estimated target number.

*SNR*_1_ and *SNR*_2_ denote the SNR of Target 1 and Target 2 respectively. When *SNR*_1_ = *SNR*_2_ = 3*dB* and the average clutter number is *λ* = 150, Figure 5 shows the recovered trajectories and the estimated target number after a single run performed. In Figure 5, “-o-” represents the recovered trajectories of the targets, and “·” represents the sensor measurements.

**Figure 6.**
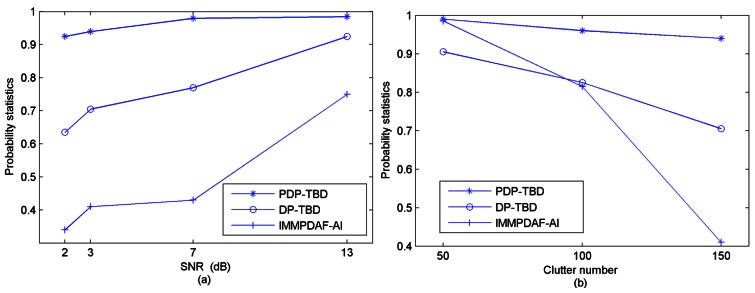
Comparison of the probabilities of correct target number estimation (200 runs, Scenario-1) (**a**) In different SNR environments (λ = 150); (**b**) In different clutter number environments (SNR = 3 dB)

The performance of PDP-TBD is compared against DP-TBD and IMMPDAF-AI for target SNR values of 13 dB, 7 dB, 3 dB and 2 dB, and for average clutter numbers of 50, 100 and 150. After 200 Monte Carlo trials are performed, Figure 6 shows the comparison of the probabilities of correct target number estimation in different SNR values and different clutter densities. When two targets are detected, Table 1 shows the comparison of correct track probabilities.

In this scenario, the two targets are well separated, hence, this scenario can be considered as a single target situation, and DP-TBD and IMMPDAF-AI can be applied to detect and track these two targets. For PDP-TBD, the tracking techniques are combined with DP-TBD through constructing a penalty term. By the penalty term, PDP-TBD has the advantages of IMM tracking methods for different target motions and the target can be more likely to be detected than the clutter. Thus, the detection performance and tracking accuracy can be improved. Inspection of Figure 6 and Table 1 verifies the efficiency of PDP-TBD in low SNR and dense clutter environment. Compared with DP-TBD and IMMPDAF-AI, PDP-TBD has better detection performance and tracking accuracy, especially when the SNR decreases and the average clutter number increases.

### 5.2. Scenario-2

**Figure 7.**
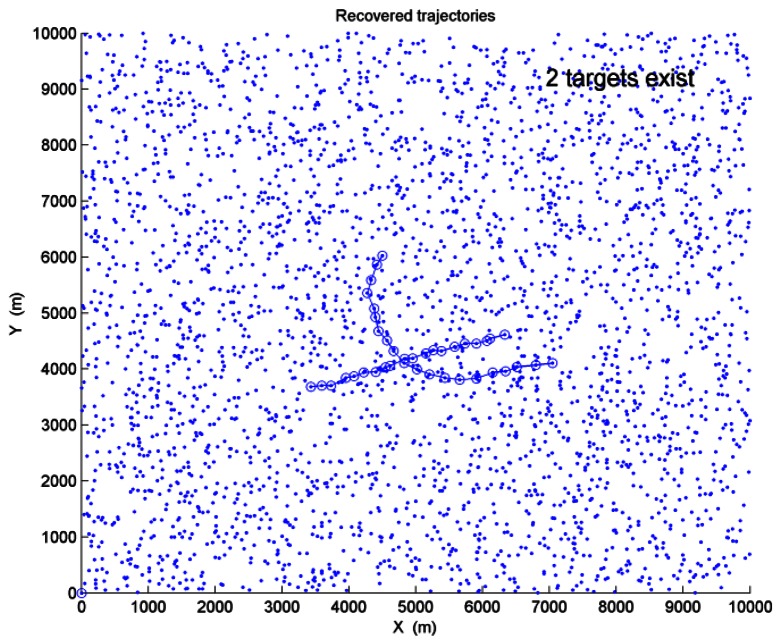
Recovered trajectories and estimated target number.

Two targets have crossing trajectories in this scenario. The target initial states are [3467,150,3684,50,0] and [4500,−100,6000,−200,0.15] with assumed positions in meter, velocities in m/s and turn rates in rad/s. Target 1 makes approximate CV motion. Target 2 makes approximate CT motion when *k* = 1 ∼ 15s, and the rest scans are approximate CV motion. The SNR values of the two targets are *SNR*_1_ = *SNR*_2_ = 3*dB* and the average clutter number is *λ* = 150. After a single run performed, Figure 7 shows the recovered trajectories and the estimated target number. In Figure 7, “-o-” represents the recovered trajectories of the targets, and “·” represents the sensor measurements.

**Figure 8.**
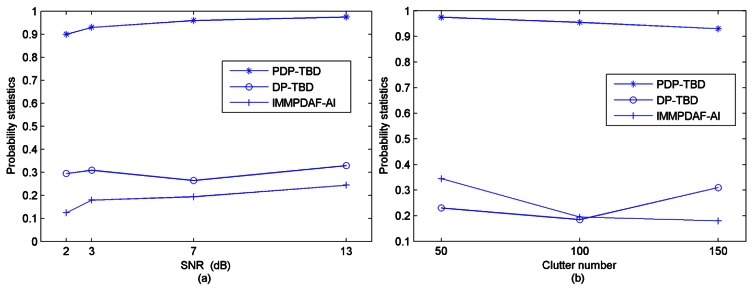
Comparison of the probabilities of correct target number estimation (200 runs, Scenario-2) (**a**) In different SNR environments (λ = 150); (**b**) In different clutter number environments (SNR = 3 dB).

After 200 Monte Carlo trials are performed, the comparison results of correct target number estimation by using these three algorithms in different SNR values and clutter densities are shown in Figure 8. When two targets are detected, Table 2 shows the comparison of correct track probabilities.

Inspection of Figure 8 and Table 2 shows that PDP-TBD is efficient to detect and track multiple maneuvering targets in this scenario, while DP-TBD and IMMPDAF-AI actually fail to detect the two targets in Scenario 2.

DP-TBD is proposed based on a single target scenario, and it performs the maximization over the state transition range in the step of recursion [5]. It is the reason which results in the track separation. The track separation has bad influence on the tracking accuracy and target number estimation. When the targets are not well separated (the targets have crossing trajectories), it is easy to lose some of the targets. IMMPDAF-AI is proposed based on a single maneuvering target tracking. In track formation and maintenance procedure, it ensures the highest probability track with the “first claim” to measurement [12]. Therefore, when using IMMPDAF-AI to deal with the targets which have crossing trajectories, it is easy to lose some of the targets. On the other hand, for PDP-TBD, a step called repetition is added to the algorithm procedure compared with DP-TBD. Meanwhile, by constructing a penalty term in the merit function, the tracking accuracy of PDP-TBD can be improved and the disadvantage by using repetition step can be greatly alleviated. With these technical modifications, the track separation can be alleviated and PDP-TBD can be applied to detect multiple targets. Furthermore, compared with Scenario 1, the two targets are not well separated, so DP-TBD and IMMPDAF-AI actually fail to detect the two targets in this scenario. By contrast, PDP-TBD is proposed based on multiple maneuvering targets scenario, so its performance is much better than IMMPDAF-AI and DP-TBD.

### 5.3. Scenario-3

**Figure 9.**
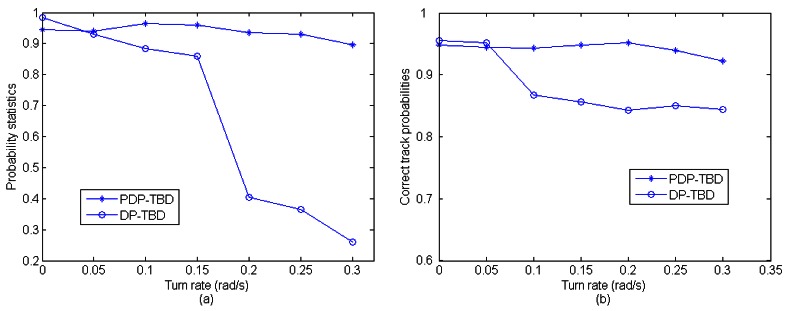
Comparison of the performances of the algorithms (200 runs) (**a**) Comparison of the probabilities of correct target number estimation; (**b**) Comparison of the correct track probabilities.

In this scenario, the performance of PDP-TBD is compared with DP-TBD in different turn rates (different speed of the target maneuvering). A target appears in the surveillance region with initial position [4500,6000] and initial velocity [−100,−200]. The target makes an approximate CT motion, and the SNR is 3 dB. The average clutter number is *λ* = 150 After 200 Monte Carlo trials, Figure 9(a) shows the comparison of the probabilities of correct target number estimation. When the target is declared, Figure 9(b) shows the comparison of correct track probabilities. According to Figure 9(a), the performance of PDP-TBD is not influenced much by the turn rate. However, when the turn rate increases, the performance of DP-TBD falls rapidly. When the target is declared, inspection of Figure 9(b) shows that the tracking accuracy of PDP-TBD is higher than DP-TBD.

### 5.4. Scenario-4

**Figure 10.**
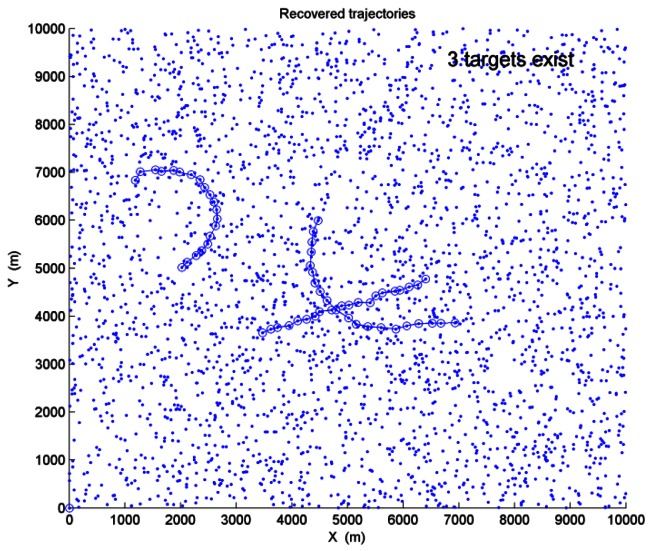
Recovered trajectories and estimated target number (Scenario-3, “-o-” represents the recovered trajectories of the targets, “·” represents the measurements).

Three targets appear in this scenario. The target initial states are [3467,150,3684,50,0], [4500,−100,6000,−200,0.15] and [2000,150,5000,100,0.12] with assumed positions in meters, velocities in m/s and turn rates in rad/s respectively. Target 1 makes an approximate CV motion. Target 2 makes an approximate CT motion when *k* = 1 ∼ 15s, and the rest of the scans are approximate CV motions. Target 3 makes an approximate CT motion. The SNR values of the three targets are *SNR*_1_ = *SNR*_2_ = 3*dB* and the average clutter number is *λ* = 150. After a single run is performed, Figure 10 shows the recovered trajectories and the estimated target number. In Figure 10, “-o-” represents the recovered trajectories of the targets, and “·” represents the sensor measurements. Then 200 Monte Carlo trials are performed. The performance of target number estimation is shown in Table 3. When three targets are detected, Table 4 shows the correct track probabilities. Inspection of Table 3 shows that PDP-TBD can be applied to efficiently detect and track more than two targets.

## 6. Conclusions

A new algorithm called PDP-TBD has been presented for simultaneously detecting and tracking multiple maneuvering dim targets with unknown target number. The algorithm uses the performances of tracking techniques as a feedback to the detection part, and the feedback is constructed by a penalty term. The penalty term is a function of the possible target state estimation, which can be obtained by the tracking methods. With this feedback, traditional tracking techniques can be combined with DP-TBD. Meanwhile, a constraint that a measurement can originate from one target or clutter is proposed to minimize track separation. With the above two technical modifications, the application scope of PDP-TBD is significantly extended compared with DP-TBD. Simulation results justified the performance of PDP-TBD under a variety of conditions.

Therefore, the proposed algorithm can be applied to simultaneously detect and track dim targets using sensor data. In practical applications, the sensor can be radar, sonar, *etc.* For example, an early warning radar system is applied to detect and track the targets. When multiple stealth aircrafts move into the radar surveillance region and the system is seriously jammed by the clutters. Using PDP-TBD to deal with these radar data, higher detection performance and tracking accuracy can be obtained compared with DP-TBD. If these aircrafts are not friendly, it is very important to detect and track them more accurately.

For PDP-TBD, the scale factor in the penalty term is affected by the measurement noise and the estimation errors. It is a constant and is empirically determined in this paper. However, the performances of traditional tracking techniques may change in different practical applications. Therefore, the scale factor should be adaptive according to the tracking performances. The Cramér Rao Low Bound (CRLB) theories may be a feasible method to estimate the tracking accuracy, it sets a lower bound on the various of any unbiased estimator [29]. Further interesting studies can be drawn to design an adaptive scale factor according to CRLB theories.

Furthermore, the constraint of a measurement having only one source is used in this paper. However, this constraint can be relaxed considering practical applications. For example, when a target moves into the surveillance region, a sensor with high resolution may provide several measurements, which are all originated from the target. And a sensor with low resolution may provide a measurement, which is actually originated from the target and clutter, *i.e.*, the clutter and target measurement are coincided. Therefore, further interests can be focused on how to design a new constraint and apply it to PDP-TBD algorithm.

## Figures and Tables

**Table 1.** Comparison of correct track probabilities (200 runs, Scenario-1).

	**Algorithm**	**SNR(λ = 150)**				**λ(SNR = 3 dB)**		

		**13dB**	**7dB**	**3dB**	**2dB**	**50**	**100**	**150**
Target 1(%)	PDP-TBD	98.20	96.25	94.79	94.00	98.46	96.33	94.79
	DP-TBD	96.95	92.95	83.76	82.24	95.14	89.55	83.76
	IMMPDAF-AI	89.17	84.65	79.15	73.90	94.70	91.72	79.15

Target 2(%)	PDP-TBD	98.96	96.86	94.36	94.62	98.41	97.11	94.36
	DP-TBD	96.95	90.94	81.21	81.18	94.61	89.82	81.21
	IMMPDAF-AI	94.33	90.70	87.13	79.19	95.23	88.80	87.13

**Table 2.** Comparison of correct track probabilities (200 runs, Scenario-2).

	**Algorithm**	**SNR(λ = 150)**				**λ(SNR = 3dB)**		

		**13dB**	**7dB**	**3dB**	**2dB**	**50**	**100**	**150**
Target 1 (%)	PDP-TBD	90.95	92.63	93.87	93.11	96.49	95.29	93.87
	DP-TBD	69.47	66.23	55.65	50.93	73.04	59.86	55.65
	IMMPDAF-AI	87.35	81.15	79.86	71.20	97.61	89.49	79.86

Target 2 (%)	PDP-TBD	90.59	91.61	91.24	91.25	95.82	93.69	91.24
	DP-TBD	61.06	63.40	51.29	55.25	69.02	63.65	51.29
	IMMPDAF-AI	78.47	63.46	75.56	35.20	93.12	86.15	75.56

**Table 3.** Probabilities of correct target number estimation (200 runs, Scenario-3).

**Estimated target number**	**0**	**1**	**2**	**3**	**others**
Probability statistics (%)	0.0	1.0	8.5	90.0	0.5

**Table 4.** Correct track probabilities (200 runs, Scenario-3).

**Target**	**No.1**	**No.2**	**No.3**
Probability statistics (%)	92.50	91.53	95.14
